# Supplementary figures and images for: Neuropeptide F signaling regulates parasitoid-specific germline development and egg-laying in Drosophila
Source: PLoS Genet. 2021 Mar 26;17(3):e1009456. doi: 10.1371/journal.pgen.1009456 (PMC8026082; doi:10.1371/journal.pgen.1009456)

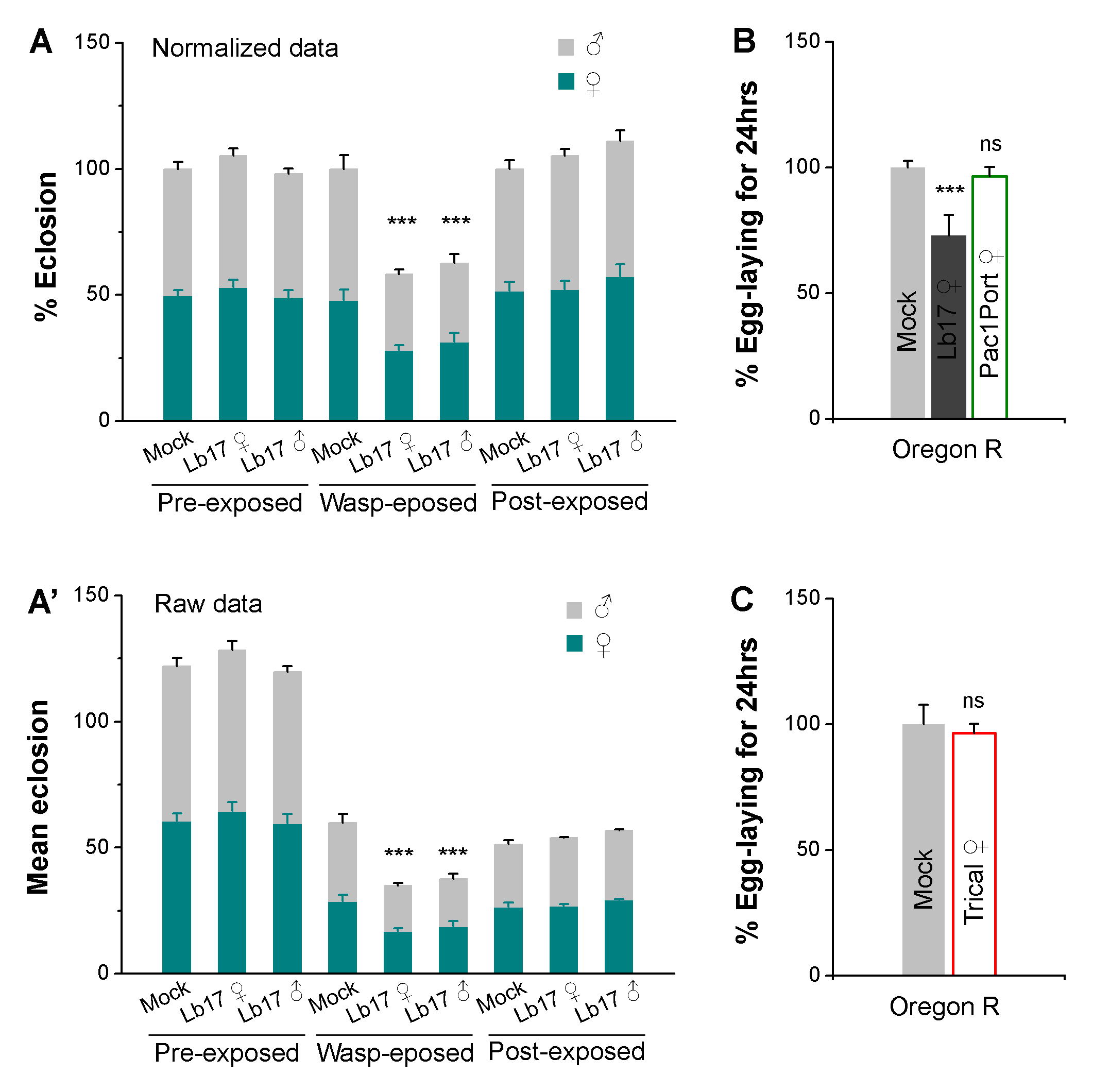

Supplement: S1 Fig — (A and A’) Stacked histogram showing the average number of female (cyan) and male (light grey) flies eclosed from mock and wasp-exposed groups: (A) normalized and (A’) raw eclosion data. (B) and (C) Histogram showing egg-lay responses of wildtype Oregon R females to larval (Lb17) and pupal (Pac1Port and Trical) parasitoids. Light grey bars correspond to egg-lay responses of mock-exposed flies. Error bars are ± SEM. *** p ≤ 0.001 and ns for non-significance (p > 0.05) calculated using Student’s t-test. (TIF) [file pgen.1009456.s001.tif]

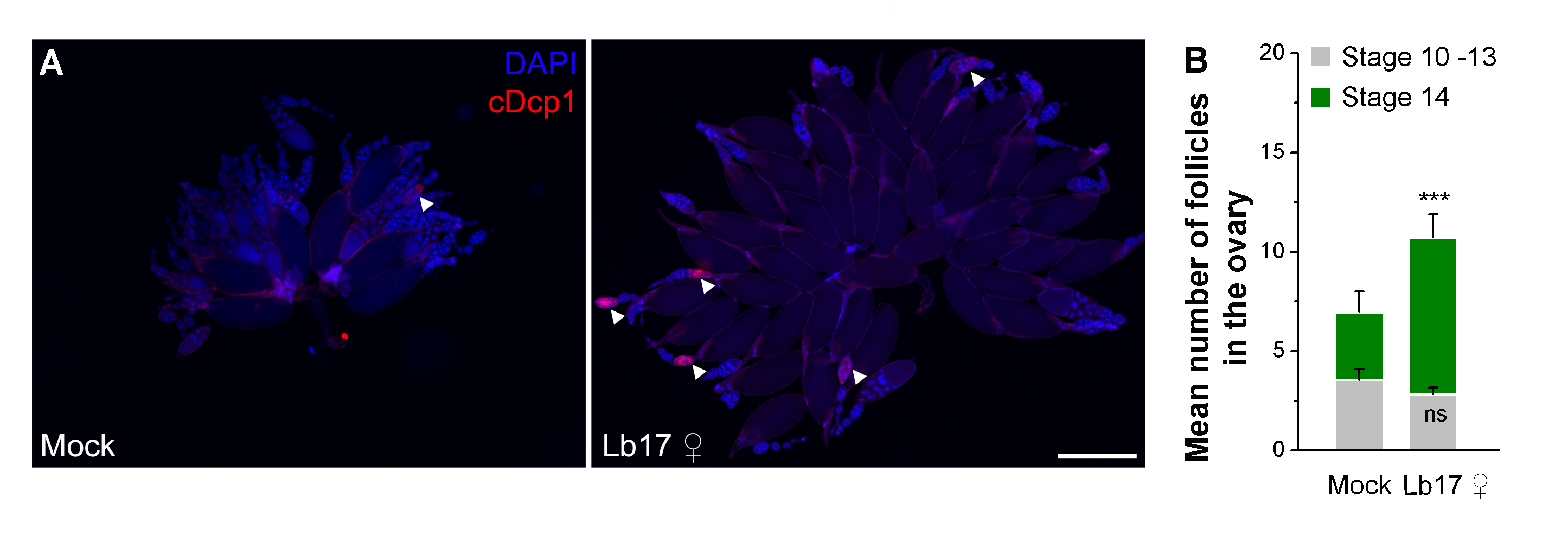

Supplement: S2 Fig — (A) Whole-mount preparations of mock and Lb17 ♀-exposed CS ovaries that are immunostained with DAPI (blue) and cDcp-1 (red). The arrowheads indicate the cDcp-1-positive egg chambers. The scale bar corresponds to 500 μm. (B) Stacked histogram showing the average number of stages 10–13 (light grey) and stage 14 (green) follicles in Oregon R female ovaries that are either exposed or unexposed to Lb17 ♀ wasps. Error bars are ± SEM. *** p ≤ 0.001 and ns for non-significance (p > 0.05) calculated using Student’s t-test. (TIF) [file pgen.1009456.s002.tif]

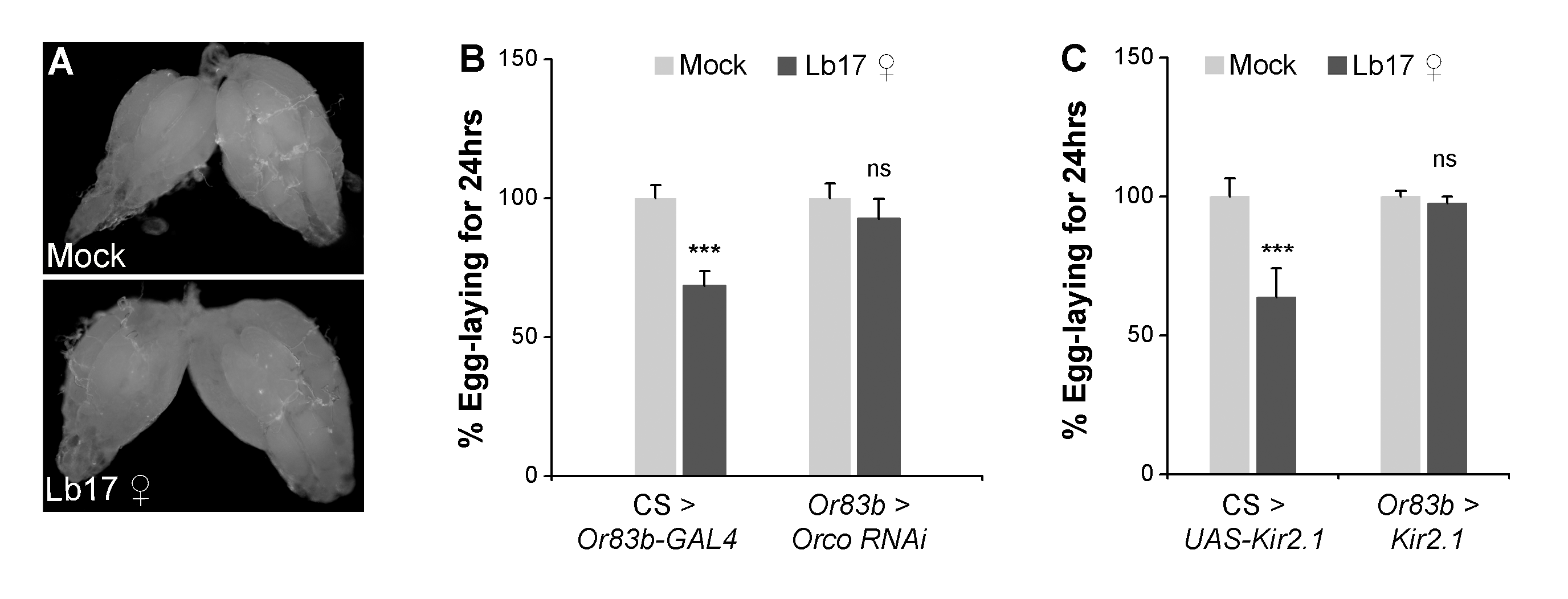

Supplement: S3 Fig — (A) Representative ovary images of CS flies that are exposed to Lb17 ♀ in the absence of light. Histogram showing the Lb17 ♀-induced oviposition behavior in flies expressing (B) UAS-Orco RNAi and (C) UAS-Kir2.1 in ORNs. Error bars are ± SEM. *** p ≤ 0.001 and ns for non-significance (p > 0.05) calculated using Student’s t-test. (TIF) [file pgen.1009456.s003.tif]

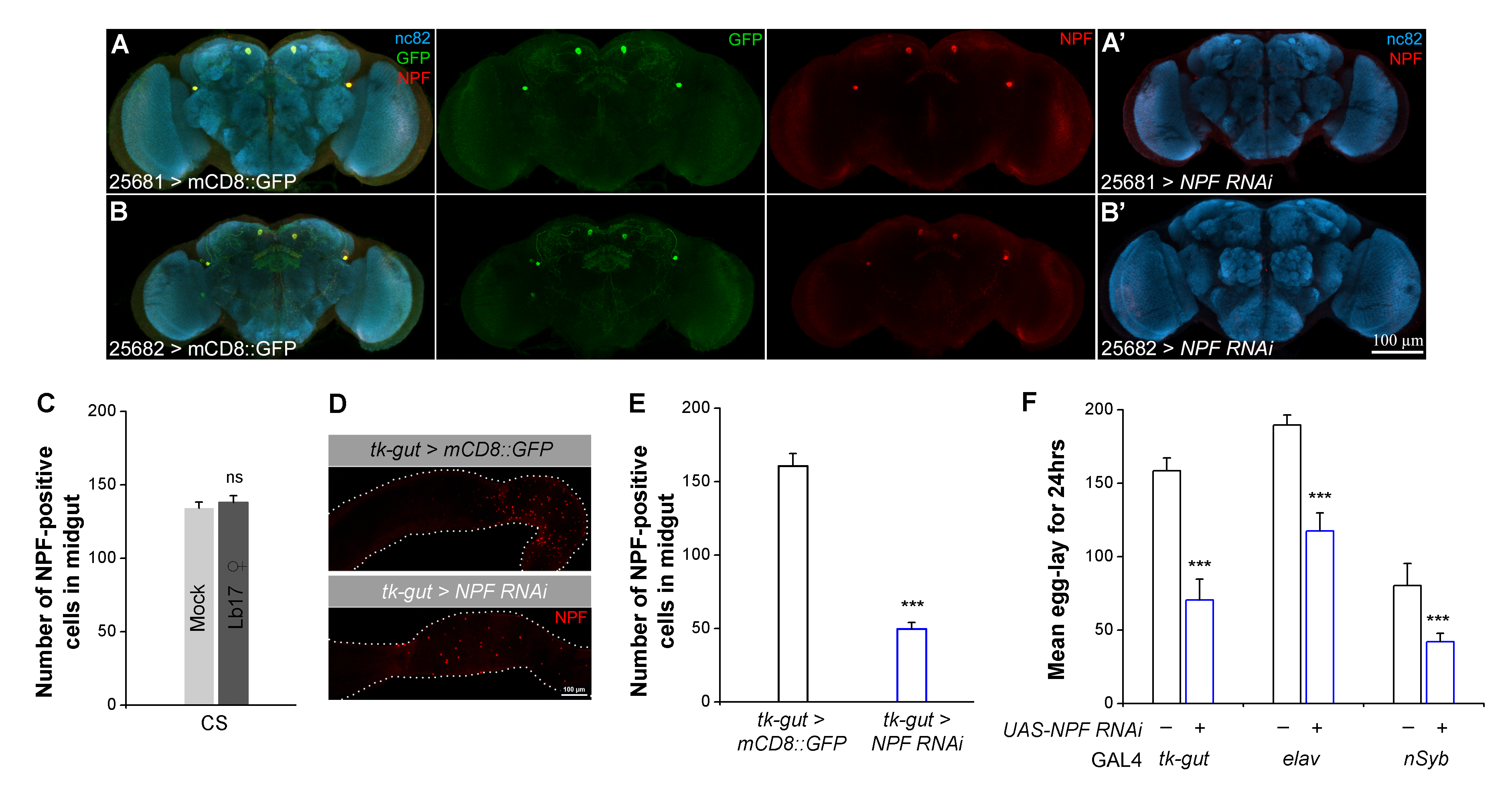

Supplement: S4 Fig — (A) and (B) Confocal images of two different NPF-GAL4 drivers expressing either (A and B) UAS-mCD8::GFP or (A’ and B’) UAS-NPF RNAi. Brains are immunolabeled with anti-GFP (green), anti-NPF (red) and anti-bruchpilot (blue). The scale bar corresponds to 100 μm. (C) The average number of NPF-positive midgut cells in LB17 ♀-exposed (dark grey) CS flies are indistinguishable from their mock controls (light grey). (D and E) tk-gut-GAL4 > UAS-NPF RNAi flies showed significant reduction of the number of NPF-positive cells in the midgut. (F) Histogram showing the average basal egg-lay in flies expressing UAS-NPF RNAi using GAL4 drivers–tk-gut-GAL4, elav-GAL4, and nSyb-GAL4. Error bars are ± SEM. *** p ≤ 0.001 and ns for non-significance (p > 0.05) calculated using Student’s t-test. (TIF) [file pgen.1009456.s004.tif]

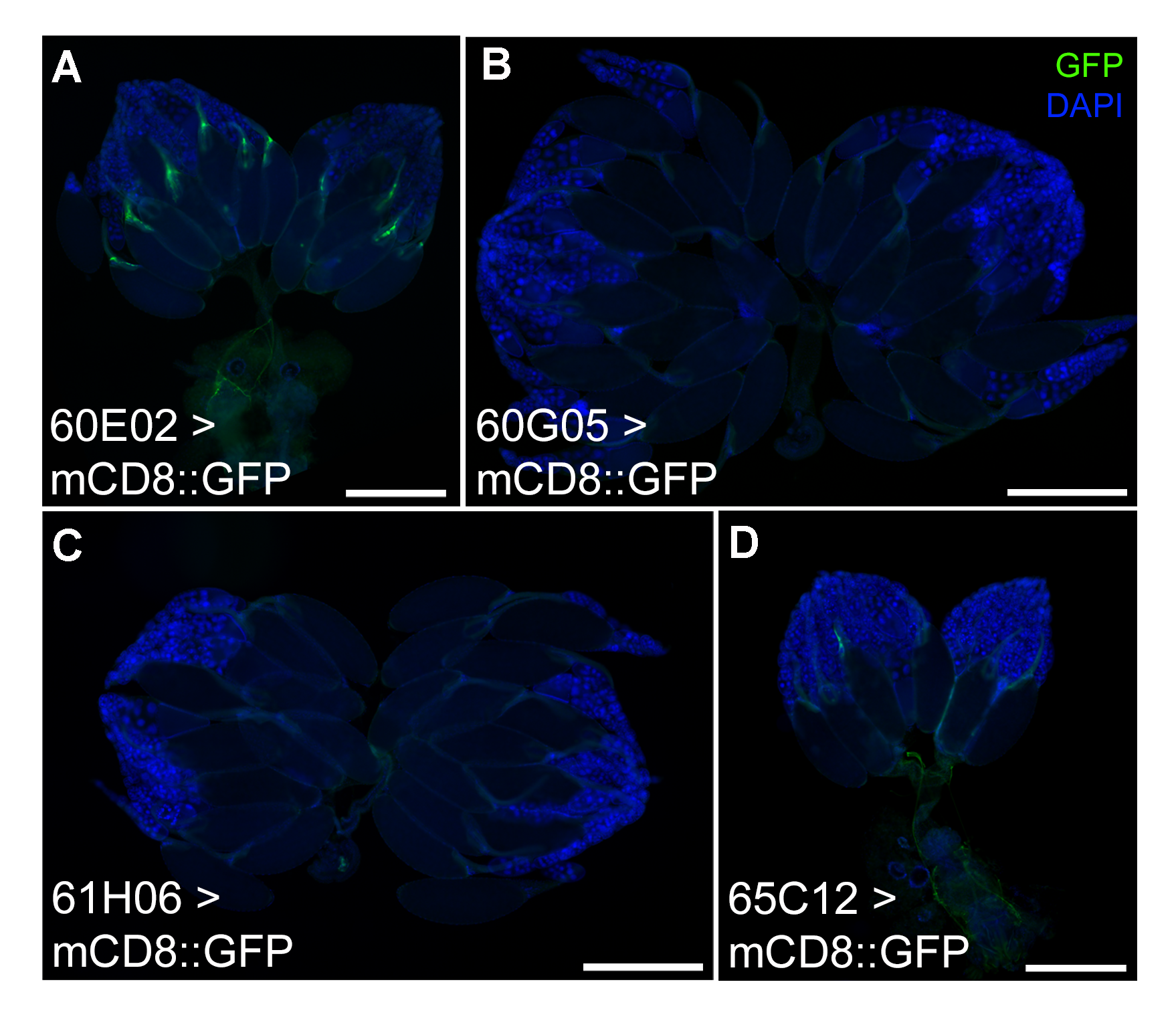

Supplement: S5 Fig — (A-D) Representative images of anti-GFP immunostaining in the ovaries of NPFR-GAL4 drives: (A) 60E02, (B) 60G05, (C) 61H06 and (D) 65C12-GAL4s expressing UAS-mCD8::GFP reporter. Nuclei are stained with DAPI. The scale bar corresponds to 500 μm. (TIF) [file pgen.1009456.s005.tif]
