# Supplementary material for: Neuropeptide F signaling regulates parasitoid-specific germline development and egg-laying in Drosophila
Source: PLoS Genet. 2021 Mar 26;17(3):e1009456. doi: 10.1371/journal.pgen.1009456 (PMC8026082; doi:10.1371/journal.pgen.1009456)
Supplement: Attachment — Submitted filename: Response to Reviewers_Sadanandappa et al.pdf [file pgen.1009456.s007.pdf]

***Reviewer #1***

*Sadanandappa et al. present a very interesting study combining Drosophila genetics with the naturally occurring phenomenon of wasp parasites that attack Drosophila larvae in high numbers in the wild. Interestingly, while some larvae manage to escape the attack, adult female flies still try to avoid egg-laying when wasps are present. How this works is not well understood and is elucidated in the present study. More generally, the study identifies a neuroendocrine mechanism that links sensory perception not only with behavior, but also with cellular physiology of an internal organ. That this axis between brain and organ is extremely important becomes increasingly clear. Nevertheless, we still know very little about it. Thus, this study is important and will be of interest to the broad readership of PlosGenetics.*

*The authors show that adult flies recognize and react to a combination of visual and olfactory cues produced by the wasp, Leptopilina, leading to a reduction in egg-laying through two different mechanisms, namely increased apoptosis in the germline and retention of maturing eggs. This effect is induced by brain-derived NPF and NPF receptors in the nervous system. By contrast, NPF in the gut or receptors outside the nervous system appear to be redundant. The authors also show that the suppression of egg-laying is independent of the mushroom body and therefore, likely, memory is not required or induced through wasp encounters. Finally, the authors speculate that sensory signals might more generally modulate cell proliferation in distant tissues.*

*I enjoyed reading the paper and would like to see it published in Plos Genetics. The data is very sound and of high quality. The authors present many genetic controls throughout the paper, which is rare and commendable. Nevertheless, I have a few suggestions that could further improve the manuscript.*

We thank the reviewer for the supportive reviews of our manuscript.

*1. The last statement in abstract and manuscript, e.g. "These observations raise the intriguing possibility that animals employ sensory-driven neuroendocrine signaling to modulate the proliferation and development of cells of distant tissues." is, in my opinion, a bit strong. While the authors show that sensory cues and NPF are required to induce germline and behavioral changes, how this works and how direct this is, remains unclear. I would recommend to remove this statement from the abstract.*

In the revised manuscript, we have removed this statement from the abstract. We have changed the discussion section and included the recent mammalian studies as suggested by Reviewer #3 (Lines 513 – 519).

*2. The authors use MB247-Gal4 to probe the role of the mushroom body. MB247 is one of the older drivers and primarily labels the α/β and γ lobes and not all types of Kenyon cells equally. While I agree that the mushroom body is likely dispensable for the observed effects, I would like to see this experiment repeated with an additional, broader driver, e.g. MB10B-Gal4.*

We agree with the reviewer that the *MB247-GAL4* driver labels a subset of mushroom body (MB) neurons, and therefore, we cannot exclude the possible function of other MB subsets in the observed behavior. As per the editor's suggestion, we have changed the text to reflect the limitations of our current data (Lines 278 - 295). We also note that the previous studies (Kacsoh *et al.,* 2015 and 2018) showed *MB247*-positive MB neuronal function in *Leptopilina heterotoma*-induced memory for egg-lay depression, which led us to examine *MB247-GAL4* in *L. boulradi*-induced innate behavioral modifications.

*3. The authors refer to the very nice publication by Ameku et al., PlosBiology 2018 that used a previously published driver, TKg-Gal4, which is supposedly expressed only in the gut but not in the brain. Unfortunately, this driver is not specific to the gut as recently stated in an erratum (DOI: 10.1016/j.celrep.2020.02.011). While this finding has no direct consequence on the presented results by Sadanandappa et al., I would still suggest to clarify this throughout the manuscript whenever the study by Ameku et al. is cited.*

We thank the reviewer for raising this point and for the reference. We have clarified this point and also cited Song *et al.,* 2020 in our revised manuscript (Lines 353 - 355).

***Reviewer #2***

*This is a very intriguing study showing that adult fruitfly females respond to presence of a specific species of parasitic wasps by diminishing egg-laying, and thereby safeguarding their offspring (for later). Based on visual and olfactory inputs the flies respond to presence of wasps by retaining mature oocytes and inducing cell specific apoptosis of developing oocytes leading to reduced egg-laying. The authors performed relevant experiments to establish the necessity of vision and specific odor input for the ovarian response to wasps. This reduced egg-laying was shown to be an innate behavioral/physiological response and not dependent on learning and activity in mushroom bodies. The authors find that signaling with neuropeptide F (NPF) is required for this integrated response to wasps. Moreover they demonstrate that NPF from brain neurons, not enteroendocrine cells of the gut, is required for retaining mature oocytes and activating apoptosis during mid-oogenesis. Unfortunately they did not manage to identify the targets of NPF underlying the reduced egg-laying. NPF receptor (NPFR) expressed in ovaries was not found to be involved, and no NPFR-expressing neurons in the brain and ventral nerve cord (VNC) could be identified as targets after a small RNAi screen.*

*A previous study has shown that after mating a gut-derived NPF acts directly on ovaries to control germ stem cell proliferation, suggesting that two mechanisms involving NPF exist that can reduce number of eggs. The present study shows that an integrated sensory input utilizes a brain neuroendocrine system to modulate egg-laying in response to presence of a wasp. Seemingly there is no crosstalk between these two pathways (i. e. non-overlapping "circuits" are employed).*

*One very general comment is that it is not always clear when authors present totally new data. They seem to repeat some experiments performed earlier, and also study NPF signaling, based on earlier studies, but they do not clearly indicate the differences between the previous and novel experiment design and subsequent findings. I will provide more detailed comments to this below.*

*In summary, it is an interesting study describing an astonishing mechanism to safeguard offspring. The neuroendocrine mechanism is also very cool, but I think the value of the paper would be improved very much by identifying the NPFR-expressing targets of the brain NPF neurons. I will provide some suggestions below.*

We thank the reviewer for this clear summary of our findings.

***Comments and suggestions***
***1.*** *Line 291: To the statements*
*"Similarly, in the presence of a Leptopilina wasp, NPF signaling facilitates the ethanol-seeking behavior in Drosophila females [5,17]." and Line 293: "A most recent study has demonstrated the role of midgut-derived NPF in mating-induced GSC proliferation in the ovaries [42]."*

*These statements are a bit vague. It would be useful if the NPF mechanisms in these three papers were described in some more detail. For instance, it is not clear in the text of the present paper what induces the NPF signaling from the gut that affects germ cell proliferation in [42] (we will find out later: it is induced by mating, so very different from parasite presence – this is revealed only on line 327 and needs to be mentioned earlier).*

We thank the reviewer for pointing out the unclear sentences. We have rephrased the text in the revised manuscript (Lines: 304 - 310) and mention the specific context in which NPF signaling is shown to function in the studies that are being cited.

*Also the NPF signaling that induced ethanol-preference should be described in some more detail – now it reads "...NPF production is increased" and "...NPF signaling facilitates the ethanol-seeking behavior...". Are these experiments very different from those in the present paper, or? What is new in the present paper?*

We have added more detail to describe the role of NPF in the behaviors (Lines: 299 - 308).

We show that both visual and olfactory inputs are required for *Leptopilina boulardi*-induced egg-lay reduction, whereas previous studies showed that only visual cues are necessary for *L. heterotoma*-induced ethanol-seeking behavior and oviposition depression (Kacsoh et al., 2013, 2015 and Bozler et al., 2019). Besides using a different wasp species, the exposure protocol employed in the current study measured the innate behavioral response of the flies, but not the learned behavioral modifications, which are retained long after wasp removal. Further, we show that NPF is required for retention of matured egg chambers and caspase-mediated apoptosis of the vitellogenic follicles resulting in egg-lay reduction in the presence of *L.boulardi*. However, *L. heterotoma* exposure reduces NPF expression that triggers only caspase-mediated apoptosis in female germline resulting in reduced egg-lay.

Given the differences in the predation strategies of wasps - *L. boulardi* is a specialist that mostly parasitizes *D. melanogaster* and *D. simulans* clade, whereas *L. heterotoma* is a generalist that infects diverse species of *Drosophila*, the mechanisms reported in the current manuscript shed light on the ecological importance and evolution of anti-predation strategies in adult *Drosophila*.

**2.** *Line 207: To the statement*
*"Since previous reports have demonstrated that the visual inputs are necessary for Drosophila to respond to the larval parasitoids [15,16], we repeated behavioral assays in the dark to test whether the same was true for the Lb17 response"*

*What was the difference to the earlier studies? Did they use different wasps? Should be clarified.*

We have clarified that the previous studies used different wasp species (Lines: 214 - 216).

**3.** *Line 272: To the statement*
*"However, a learned response that involves a memory component appears to be mediated by mushroom body (MB) neurons [16], which are known to integrate and process different sensory modalities [34–36]."*

*Is this also a response to wasps? If, so it would be valuable to get information about how this response differs from the one in the present paper. Based on line 280, the study [16] also involves reduced egg-laying.*

Kascoh *et al.,* 2015 reported the learned egg-lay depression, which persisted long after *L. heterotoma* wasp removal, and this response requires the function of *MB247*-positive mushroom body neurons. However, our study examined the innate behavioral response - in the presence of *L. boulardi*, but not the learned behavioral modifications - in the absence of wasps.

As opposed to the previous studies that sought to elucidate the genetic basis of persistent behaviors,

such as oviposition preferences, oviposition depression, and transgenerational effects, our study addressed how parasitoid-selective sensory input affects germline development and physiology. We rephrased the text to state the focus of the current study (Lines: 278 - 295).

***4.*** *Line 281: Associated with the comment above, it should be pointed out that MB247 positive neurons is only a subpopulation of the MB neurons, and that theoretically other populations could mediate a learned behavior.*

We thank the reviewer for raising this point, as pointed by Reviewer #1. As addressed above, we have changed the text to reflect the caveat that the *MB247-GAL4* driver does not express in all MB neurons (Lines: 278 - 295), and therefore we can't exclude the possible function of other subpopulation MB neurons in the observed innate response to *L. boulradi*.

***5.*** *Line 357: To the statement*
*"However, NPFR is also expressed in non-neuronal tissues, raising the possibility that brain-derived NPF signaling targets non-neuronal cells."*

*It is important to note that as far as I know (based on antisera and GAL4-drivers) NPF in the brain is only expressed in interneurons, so all targets of brain NPF neurons should reside within the brain or ventral nerve cord (VNC) where axons from descending NPF neurons project. NPFR outside the CNS should respond to hormonal NPF from gut EECs only. Thus it is not surprising if an ovarian NPFR is not involved. In any case the NPF targets of the CNS are yet to be identified.*

We thank the reviewer for this comment. In the revised manuscript, we have deleted the above statement and restated the ovarian NPFR observation in lines: 389 - 391.

***6.*** *Line 380: The screen for NPFR-expressing neurons that could mediate the ovarian response was not exhaustive, and the statement on line 407 a bit unfortunate: "Thus, we cannot exclude the possibility that 60G05-GAL4 driven NPFR depletion perturbs ovarian NPFR expression in an undetectable but significant manner and/or alters NPFR expression in cells that are yet to be described."*

*This sentence also needs rephrasing while "in an undetectable but significant manner" sounds weird (you do detect a significant manner!).*

We thank the reviewer for pointing out this confusing sentence. Our intent to convey - though 60G05-GAL4 driven *UAS-mCD8::GFP* signal were not detected in the ovarian cells, the expression of *UAS-NPFR RNAi* might still have significant biological effects in the ovary. We have simplified this sentence, and it is now in line 429 - 431.

***But importantly***, *I would suggest considering a few putative NPFR-expressing cell systems that could mediate the NPF action to ovary cells.*

***Strategy 1*** *would be to use trans-tango labeling (or GRASP) to identify all NPF target neurons, since it appears that the NPRF-GAL4 lines are variable and incomplete.*

***Strategy 2*** *would be to screen other putative target cells that could express the NPFR.*

*The NPFR is functionally expressed on insulin-producing cells (IPCs) in the pars intercerebralis. ILPs could target the ovaries as hormone. Surprisingly the NPFR GAL4 lines used in the present study do not include the IPCs.*

We agree with the reviewer on these experimental suggestions. The role of insulin-like peptides (ILPs) in Lb17-induced behavioral and germline modification is certainly an area that we plan to explore next.

Intending to identify the downstream target of NPF signaling, we tested all available *NPFR-GAL4* lines and also performed RNAi-mediated NPFR knockdown using a multitude of GAL4-drivers expressed in germline cells, follicle cells, ellipsoidal body, and other tissues (Fig. 7 and 8). Unfortunately, we could not specifically identify the subset of NPFR expressing cells that respond to brain-derived NPF. We feel that the reviewer's proposed strategies will be excellent approaches for our future direction of the study, and therefore, outside the scope of the present manuscript. As per the editor's suggestion, we have not presented any additional experiments to identify the NPF targets in the current study.

*Does the 60G05-GAL4 show up in any neurons with axons reaching outside the CNS? It is hard to tell from the images provided. There are several types of neurons in the abdominal ganglia (in addition to Tdc-GAL4 neurons) that have efferent axons. Maybe some of these innervate the ovaries or have axon terminations in the periphery from which a hormonal substance could be released to reach the ovaries. For example neurons producing:Pigment-dispersing factor, Crustacean cardioactive peptide, Ion transport peptide, RYamide, Allatostatin A, Proctolin, ILP7*

60G05-GAL4 > *UAS-mCD8::GFP* flies showed reporter expression in the thoracic ganglia neurons (please refer to Fig. 9A), but not in the ovaries (Supplementary Fig. 5B). Our attempts to analyze the direct contact between 60G05-GAL4 expressing cells with ovarian tissues were unsuccessful. However, 60G05-GAL4 > *UAS-mCD8::GFP* flies did not reveal any visibly labeled neuronal remnants in the ovaries.

*Also neurons expressing the ILP8 receptor Lgr3 (in abdominal ganglia) seem to innervate ovaries (Lgr3VP16-Gal4, from M Texada)*

The role of ILPs, their receptors, and the specific cell-types that may mediate the germline modification in response to wasp is of great interest to us that we plan to explore next. However, we feel that such experiments are outside the scope of the current study.

***To Discussion***

***7.** On Line 450: To the statement*
*"A most recent study showed that parental exposure to parasitoid wasps leads to depression of NPF-signaling in the adult fly brain, which triggers caspase-mediated germline apoptosis and ethanol preferences."*

*Is this analysis of germ-line apoptosis any different from the one presented in the present paper? How is the regulation of ethanol seeking/preference coming about (what circuits/pathway)? Would be useful to get some more information here.*

This sentence has been rewritten and is now at lines 475 - 481. Briefly, as mentioned before flies were exposed to *L. boulardi* for 24 hrs in our study, whereas in Bozler *et al.,* (2019) study, flies were exposed to *L. heterotoma* for 4-days. Besides, these differences in exposure protocols, the observed egg-lay depression in our experiments require both visual and parasitoid-specific olfactory inputs, whereas the ethanol-seeking behavior necessitates only visual cues.

***8.** On Line 470: To the statement*

*"The recruitment of NPF-NPFR signaling requires parasitoid-specific visual and olfactory cues, and the NPF-responsive central region that integrates these sensory inputs is yet to be identified."*

*Can the authors speculate which region is important? Commonly the so-called lateral horn (LH) of the brain is a target of pheromone-like signals and also receives visual input. In Fig. 5 one can see NPF expressing branches in the LH (upper left and right corners of image). Presumably both NPF cell types shown in Fig 5 have branches in the LH (and elsewhere).*

We agree with the reviewer that NPF is expressed in the higher brain centers including, the lateral horn (LH), mushroom body, and the central complex (Fig. 5A). These brain centers play a crucial role in sensory integration, motor control, sleep, innate behaviors, and learning and memory. As the reviewer rightly pointed out, in addition to processing and integration of the sensory modalities, the LH also mediate innate behavioral response in insects. LH neurons could be a potential target for NPF signaling, which needs to be investigated.

***9.** On Line 473: It is not made clear to the readers that the study [42] describes a very different pathway.*

*"NPF expression in the wasp-exposed fly brain functions to modify oviposition preferences [5,17], whereas the mid-gut expressed NPF controls GSCs proliferation [42]."*

*Can the authors reveal under which conditions gut-derived NPF acts on ovaries to make clear it is very different from the wasp-induced signaling? The paper [42] describes NPF signaling induced by mating (which is a very different pathway).*

We have clarified this sentence in the revised manuscript, and it is now at lines 500 - 503.

***10.*** *On Line 476: To the statement*
*"Given the conserved function of NPF and its mammalian ortholog NPY in regulating stress responses [60–62], these questions are of fundamental relevance to both behavioral immunity and neuromodulation of germline physiology."*

*This is too simplified. NPY/NPF also regulates feeding, metabolism, aggression, mating and clock functions, so one may also see an influence of altered NPF signaling on other behaviors and metabolism after exposure to a wasp. As the authors mention, stress may reallocate resources for ovary development (and fecundity) to somatic maintenance (like in diapause). Guess also sleep, feeding and receptivity to males would alter when a wasp appears. That would mean orchestration of behavior-metabolism-physiology for the purpose of protecting offspring (delaying offspring) when exposed to a wasp. This could mean that wasp-induced NPF targets many more systems than ovaries.*

We thank the reviewer for this explanation. We strongly agree with the reviewer that the presence of wasps alters various behavioral, metabolic, and physiological processes both in larvae as well as adult flies. Given the known function of NPF in insects, we will not be surprised that wasp-induced NPF signaling targets many more systems than ovaries, and elicits various behavioral modifications, which needs to be examined. We believe that future studies will report an increasing number of evidences in support of this idea.

***11.*** *On Line 487: To the statement/conclusion*
*"For example, since proliferation and differentiation of adult stem cells is critical for tissue maintenance, including adult neurogenesis in mammals, it is intriguing to think that sensory inputs can modulate neuronal stem cell proliferation or differentiation. Although highly speculative at this time, if adult neurogenesis were to be continually modulated by sensory signals, then it would have broad implications for how we understand adult brain functions and maintenance."*
*This is indeed speculative (but God's ways are mysterious!).*

As stated in our response to Reviewer #1, we have revised this section and discussed the recent mammalian reports suggested by Reviewer #3 (Lines: 513 - 519).

***Reviewer #3***

*The ability of organisms to protect themselves from parasites is important for the survival of species. In nature, the vasp majority of Drosophila larvae are parasitized by wasp species, the most common being Leptopilina boulardi (a more specific parasite) and Leptopilina heterotoma (a generalist that infects many Drosophila species). Various behavior responses to evade these parasites have evolved in Drosophila, including larval rolling behavior or adult changes in egg laying and alcohol-seeking behavior for oviposition (both aimed presumably at reducing the number of future larvae exposed to the parasite). In this study, Sadanandappa et al. investigate the mechanisms underlying Drosophila melanogaster egg laying changes in response to Leptopilina parasitoids using a combination of genetic approaches and microscopy. The authors report that olfactory and visual cues are required for reduction of egg laying in response to Leptopilina, and that death of vitellogenic follicles and accumulation of stage 14, mature oocytes are part of the cellular mechanisms involved in reducing the number of eggs laid. They also report that Drosophila brain-derived neuropeptide F (NPF) signaling is required for the increased death of follicles, accumulation of mature oocytes, and reduction in egg laying in response to exposure to Leptopilina.*

***Critique****: The manuscript is clearly written, the study addresses a fascinating area of biology, and many of the key conclusions are well supported by the data. For example, experiments using mutants and RNAi knockdown convincingly demonstrate a role for visual and olfactory cues, as well as for NPF signaling in the egg-lay response of Drosophila to Leptopilina. The data showing that NPFR is required in 60G05-expressing neurons (using both NPRF RNAi and UAS-Kir2.1) are strong. The data showing death of vitellogenic follicles in response to exposure to Leptopilina (and that it depends on NPF signaling) are also convincing. However, some of the data in the manuscript are less convincing. For example, there are concerns with the quantification of follicles of different stages per ovary, and also regarding the data on the specific source of NPF required for the response to Leptopilina (see below for details). Some other clarifications are also needed, as outlined below.*

We appreciate the overall positive remarks by the reviewer.

***Major Points:***

*- In all egg count graphs, the authors should show the actual average number of eggs laid per female instead of setting the control to 100%. In figure S1A, it is unclear why the egg counts of mock exposed females go down; it would be helpful if the authors could comment on possible reasons.*

There are two important points raised by the reviewer. First, the depiction of the egg count data as a percent or raw egg numbers. In the current manuscript, we have presented the data in both ways.

The graphs are normalized to the mock exposed and thus are displayed as percent of unexposed controls. Raw egg counts for all corresponding graphs are listed in Table 1, along with the sample size and *p*-values.

Second, we would like to clarify that the data presented in Fig. S1A corresponds to of eclosion of adults (not eggs). Nevertheless, the reviewer raises an important point: There is a reduction in eclosion of mock flies in the wasp-exposed 24 hrs period, relative to their pre-exposed 24 hrs period. This observation is easily explained: Before setting up the experiments, we perform 5-days of aging in food bottles (approximately 80 to 100 female and male flies) at 25ºC in order to ensure maximum mating and fertilization. For setting up the experimental vials, we anesthetize the flies with $CO_2$ and place the flies (5 females and 2 males per vial) on a fresh media containing yeast. The transition from population-dense bottles to vials with yeast-rich food, exposure to $CO_2$, and low population density in vials, induce a bolus dump of eggs laid on the first day (pre-exposed, 0 - 24 hrs interval). However, on the second day (wasp-exposed, 24 - 48 hrs), when flies are transferred to a fresh vial without exposure to $CO_2$ anesthetization, females depleted of their stockpile of mature follicles in the previous 24 hrs, effectively have reduced egg-lay counts in the 24 - 48 hrs interval, and the effect of this depletion persists into the subsequent post-exposed 24 hrs interval (48 - 72 hrs) (since females have not had time to ramp-up follicle production). In Fig. S1A (panel A') the raw mean number of adult eclosion reflects this decrease in egg-laying for those 24 - 48 and 48 - 72 hrs intervals of the experiment. Therefore, in the revised figure, we now depict the data in two ways: First, in Fig.S1A we set each of the three-time interval mock control set at 100% eclosion, and then normalize the other groups as a percent of mock for each time interval. Second, in Fig. S1A' we show the raw data illustrating the mean eclosion for each group of flies for different time intervals. The decrease in eclosion of the mock group of wasp-exposed (24 - 48 hrs) and post-exposed (48 - 72 hrs) noted by the reviewer is apparent in the raw data. We emphasize that despite this decrease in the 24 - 48 hrs mock group, we observe a significant reduction in Lb17-exposed groups for the 24 - 48 hrs interval. We have clarified different time intervals in the methods (lines: 570 - 578), and we have sought to better explain these results in lines 133-150 of the revised manuscript.

*- The authors should clarify in the methods what the time points are for pre-exposure and post-exposure egg counts. Similarly, it is not clear in lines 144-146 how the quantification for "mean egg-lay responses for 72 hrs" was done.*

We have clarified different time intervals in the methods (lines: 570 - 578), result section (lines: 148 - 150) and Fig. 1B.

*- In multiple graphs along the manuscript (Fig. 2E, 3B,E, 4B, 5E, 6F, 9B, S2B), the authors show quantification of the number of follicles at different stages of development per ovary. Given that it would not be trivial to do this precisely in whole ovaries, the authors should explain in detail how*

*this was accurately done. Adding to this concern, the quantified numbers of stages 10-13 per ovary seem exceedingly low in these graphs. (Specifically, each ovariole under ideal conditions would be expected to have 1 or 2 of those stages, and each ovary has approximately 15 ovarioles, such that controls would be expected to have about 15-30 of those stages per ovary. Yet, in most graphs the control numbers are much lower - this is very puzzling.)*

We thank the reviewer for raising this concern. We have clarified the relevant methods sections to better describe how staged follicles were counted (Lines: 618 - 622).

As for the low numbers of stages 10-13 per ovary, it is important to note that in all experiments, we always used mock controls of the corresponding/similar genotypes so that we can directly compare any changes for given genetic background, food batch effects, etc. Additionally, (a) the ideal/expected numbers of 10-13 follicles mentioned by the reviewer may apply to young 3-5-day-old flies under ideal conditions and only in some genetic backgrounds. For example, in Oregon-R wild-type strains the Calvi and Montooth groups have shown that some ovarioles are completely devoid of stages 1-7 follicles in young 3 days-old females (Zhang et al., 2017. PMID: 28576772). Although this study did not specifically count stages 10-13, it is reasonable to infer that in subsequent days (e.g. 4-7 days) stages 10-13 would similarly be depleted as they continue to develop into mature stage 14 egg chambers; and (b) it is our experience from extensive ovary analysis of flies in many genetic backgrounds that even under ideal conditions stages 11-13 follicles are exceedingly rare (e.g. <1 per ovariole), normally found in young females 3-5 days-old and highly dependent on the type of food, temperature, and crowding, among other variables. This is mainly due to the rapid rate of developmental transition through stages 11-13, as previously documented (Spradling, 1993). In our experience, freshly eclosed females need to be raised at 18$^{o}$C or room-temperature to slow development through stages 11-13 to observe these stages in 4-6-day old females. Our experiments were all done at 25$^{o}$C in females that were older than 6 days. Similarly, in our experience stage 10 follicles in females that are older than 6 days raised at 25$^{o}$C are rare. Since we always had control/mock groups of the same genetic background analyzed at the same time and conditions as their respective experimental wasp-exposed groups, we believe that the observed differences are valid (even if the number of stage 10-13 follicles are lower than may be expected).

*- In Fig. 2 and S2, the authors measure the number of GSCs, but the rationale for looking at GSC number (instead of proliferation, for example) over a 24-hour period is not clearly explained. Also, even if they examined proliferation, any changes in GSC proliferation would not translate into an immediate change in the number of eggs produced, since it takes close to 10 days from a GSC division to a mature egg. On a related note, in lines 188-190, the authors state: "These data indicate that 24 hrs of Leptopilina exposure appears to have no long-lasted effect on GSCs proliferation." However, they did not measure proliferation of GSCs, they only measured GSC*

*numbers, which are not affected by their proliferation rates (i.e. differences in GSC proliferation alter the number of cystoblasts produced, but not the number of GSCs, given that each division of a GSC generates one GSC for self-renewal and one cystoblast for differentiation). Related to that, in line 51, it might be more appropriate to refer to survival instead of proliferation.*

We thank the reviewer for correcting our oversight, and we completely agree with the reviewer's point. The data presented in Fig. 2F correspond to the quantification of GSC numbers in mock and wasp-exposed flies, but not the proliferation of GSC. We have rephrased the text in the revised manuscript, and it is now in lines: 191 - 196.

*- It would be interesting to know if NPF levels are altered in response to visual and olfactory inputs from Leptopilina exposure (e.g. by comparing NPF levels using antibodies used in the manuscript between wildtype versus genetically manipulated flies with impaired vision or olfaction exposed to Leptopilina or mock exposed.)*

It is a very interesting experiment. There are two technical challenges in this immunohistochemistry experiment: First, the expression of NPF in the cell bodies is relatively higher compared to neurites. The imaging acquisition settings used to capture the neurite signaling saturates the NPF signals from the cell bodies. Conversely, the acquisition settings used to acquire cell body expression completely weakens the NPF signals from the neurites. Our careful attempts (including different fixation protocols and staining procedures used for sample preparation) to quantify NPF expression in wildtype mock and *Leptopilina* exposed brains were neither consistent nor convincing.

Secondly, as the reviewer noted below, marked reduction of NPF expression in *NPF-GAL4; nSyb-GAL80 > UAS-NPF RNAi* is sufficient to induce behavioral responses to Lb17. We think that the wasp exposure protocol including, short exposure duration and the wasp, *L. boulradi*, employed in our study, might induce a minimal change in NPF expression, unlike the previous studies, which used prolonged 4-day wasp exposure protocol and different wasp strain (*L. heterotoma*) that induced noticeable changes in NPF expression. Given these technical challenges, by expressing a photoconvertible protein, CaMPARI, in NPF neurons, we made an (unsuccessful) attempt to visualize the NPF neuronal activity in mock and wasp-exposed flies.

*- Fig. S4D,E: the authors should clarify if the tk-gut driver is expressed in just a subset of enteroendocrine cells (i.e. this would help explain why so many NPF-positive cells are left in the gut after NPF knockdown using this driver).*

We have clarified this in the revised manuscript, and it is now at line 351 - 353.

*- Fig. 6C and lines 343-346: there is a marked reduction in NPF levels even though the authors state they used NPF-Gal4 combined with nSyb-Gal80, which is puzzling. In any case, the fact that NPF levels are very reduced but the response to Leptopilina appears to be fully intact significantly weakens the authors' conclusion that "neuronally-expressed NPF seems to mediate the Lb17 (...) responses." (Could there be another non-neuronal, non-gut source of NPF?) The authors should use a neuronal driver (e.g. nSyb-Gal4) to knock down NPF in the brain to more conclusively test if NPF is indeed required in neurons for the response to Leptopilina.*

We agree with the reviewer on this observation. For an unknown reason, we observed a reduced level of NPF expression in *NPF-GAL4; nSyb-GAL80 > UAS-NPF RNAi*, which was sufficient to induce behavioral and germline modifications to Lb17.

As the reviewer suggested, we used the pan-neuronal drivers – *elav-GAL4* and *nSyb-GAL4* to perform NPF knockdown experiments. Unfortunately, both genotypes: *elav-GAL4> UAS-NPF RNAi* and *nSyb-GAL4 > UAS-NPF RNAi* showed a significant reduction in the basal egg-lay compared to controls. Given the inconsistency in the basal egg-lay, we couldn't measure the wasp exposure effect on egg-lay behaviors. We present theses new basal egg-lay data for *elav-GAL4> UAS-NPF RNAi* and *nSyb-GAL4 > UAS-NPF RNAi* in supplementary figure 4F and corresponding text in lines: 355 - 357.

*- Lines 361-362 and Fig. 7a: standard UAS constructs are very poorly (if at all) expressed in the female germline, so the authors cannot conclusively rule out a possible requirement for NPFR in the germline based on those experiments. (UASp or UASz transgenes are required for effective expression in the female germline.)*

We agree with the reviewer that UASt constructs effectively function in the somatic cells but not in the germline cells. Though we used two different sources of validated *UAS-NPFR RNAi* lines (VDRC KK and TRiP line in the VALIUM 10 vector) to perform the NPFR knockdown, we conclusive don't rule out function of the ovarian NPFR in Lb17-induced germline modifications (referring to lines: 387 - 389).

We also note that as mentioned by Reviewer #2 (point 5), brain expressed NPF targets the neuronal NPFR expressed in the CNS and the ventral nerve cord. However, the ovarian NPFR is mostly activated by the hormonal NPFR produced by the midgut EECs, which is dispensable for Lb17-induced behavioral and germline modifications. The potential targets of NPF signaling, *60G05*-positive NPFR expressing cells resides, in the brain and the VNC. These findings together hint that wasp-induced germline modifications may not be mediated through ovarian NPFR.

*- Lines 487-492: in the discussion, instead of just raising it as a possibility based on their study, the authors should briefly discuss existing evidence that distant neuronal inputs, including sensory*

*inputs, can indeed influence mammalian neurogenesis (e.g. see reviews: Obernier & Alvarez-Buylla, Development 2019, and Ryu et al. Molecular Brain 9:43, 2016).*

We thank the reviewer for this suggestion. We have discussed these papers in our revised manuscript (lines: 513 - 519).

*- in the Methods, more details should be including when explaining immunostaining procedures. Alternatively, reference citations could be included instead, if these procedures have already been explained in detailed previously.*

We the revised immunostaining protocol and also included the reference citations.

***Minor Points:***

*- instead of using "mid-oogenesis stages" through the manuscript, it would be more accurate to refer to those as vitellogenic follicles because more than half of the actual time of oogenesis (from GSC to stage 14) is actually spent in the germarium (e.g. see Lin & Spradling 1993, and Margolis & Spradling Development 1995).*

We thank the reviewer for correcting us. In the revised manuscript, we have replaced '*mid-oogenesis stages*' with '*vitellogenic follicles.'*

*- reference citations for genetic tools used in the manuscript are missing in multiple cases (e.g. lines 238-239; lines 280-281; line 334; line 374; also in methods).*

We have included the reference citations.

*- throughout graphs showing stages 10-13 and 14 quantification, Y-axis should refer to number of follicles (not eggs) in the ovary.*

We have changed the Y-axis label of all the graphs showing different stages of follicles quantification (Fig. 2E, 3B, 3E, 4B, 5E, 6F, 9B and S2B).

*- there is a typo in Figure 9C (Fold-change).*

We thank the reviewer for catching the error. We have corrected the typo (Fig. 6G and 9C).

*- another suggested keyword: oogenesis*

We thank the reviewer for suggestion of the keyword, which is now included in the manuscript.
