# Supplementary material for: Neuropeptide F signaling regulates parasitoid-specific germline development and egg-laying in Drosophila
Source: PLoS Genet. 2021 Mar 26;17(3):e1009456. doi: 10.1371/journal.pgen.1009456 (PMC8026082; doi:10.1371/journal.pgen.1009456)
Supplement: Attachment — Submitted filename: Review P Genetics Dec 2020.docx [file pgen.1009456.s006.docx]

This is a very intriguing study showing that adult fruitfly females respond to presence of a specific species of parasitic wasps by diminishing egg-laying, and thereby safeguarding their offspring (for later). Based on visual and olfactory inputs the flies respond to presence of wasps by retaining mature oocytes and inducing cell specific apoptosis of developing oocytes leading to reduced egg-laying. The authors performed relevant experiments to establish the necessity of vision and specific odor input for the ovarian response to wasps. This reduced egg-laying was shown to be an innate behavioral/physiological response and not dependent on learning and activity in mushroom bodies. The authors find that signaling with neuropeptide F (NPF) is required for this integrated response to wasps. Moreover they demonstrate that NPF from brain neurons, not enteroendocrine cells of the gut, is required for retaining mature oocytes and activating apoptosis during mid-oogenesis. Unfortunately they did not manage to identify the targets of NPF underlying the reduced egg-laying. NPF receptor (NPFR) expressed in ovaries was not found to be involved, and no NPFR-expressing neurons in the brain and ventral nerve cord (VNC) could be identified as targets after a small RNAi screen.

A previous study has shown that after mating a gut-derived NPF acts directly on ovaries to control germ stem cell proliferation, suggesting that two mechanisms involving NPF exist that can reduce number of eggs. The present study shows that an integrated sensory input utilizes a brain neuroendocrine system to modulate egg-laying in response to presence of a wasp. Seemingly there is no crosstalk between these two pathways (i. e. non-overlapping “circuits” are employed).

One very general comment is that it is not always clear when authors present totally new data. They seem to repeat some experiments performed earlier, and also study NPF signaling, based on earlier studies, but they do not clearly indicate the differences between the previous and novel experiment design and subsequent findings. I will provide more detailed comments to this below.

In summary, it is an interesting study describing an astonishing mechanism to safeguard offspring. The neuroendocrine mechanism is also very cool, but I think the value of the paper would be improved very much by identifying the NPFR-expressing targets of the brain NPF neurons. I will provide some suggestions below.

**Comments and suggestions**

**1.** Line 291: To the statements

“Similarly, in the presence of a Leptopilina wasp, NPF signaling facilitates the ethanol-seeking behavior in Drosophila females [5,17].” and Line 293: “A most recent study has demonstrated the role of midgut-derived NPF in mating-induced GSC proliferation in the ovaries [42].”

These statements are a bit vague. It would be useful if the NPF mechanisms in these three papers were described in some more detail. For instance, it is not clear in the text of the present paper what induces the NPF signaling from the gut that affects germ cell proliferation in [42] (we will find out later: it is induced by mating, so very different from parasite presence – this is revealed only on line 327 and needs to be mentioned earlier).

Also the NPF signaling that induced ethanol-preference should be described in some more detail – now it reads “…NPF production is increased” and “…NPF signaling facilitates the ethanol-seeking behavior…”. Are these experiments very different from those in the present paper, or? What is new in the present paper?

**2.** Line 207: To the statement

“Since previous reports have demonstrated that the visual inputs are necessary for Drosophila to respond to the larval parasitoids [15,16], we repeated behavioral assays in the dark to test whether the same was true for the Lb17 response”

What was the difference to the earlier studies? Did they use different wasps? Should be clarified.

**3.** Line 272: To the statement “However, a learned response that involves a memory component appears to be mediated by mushroom body (MB) neurons [16], which are known to integrate and process different sensory modalities [34–36].”

Is this also a response to wasps? If, so it would be valuable to get information about how this response differs from the one in the present paper. Based on line 280, the study [16] also involves reduced egg-laying.

**4.** Line 281: Associated with the comment above, it should be pointed out that MB247 positive neurons is only a subpopulation of the MB neurons, and that theoretically other populations could mediate a learned behavior.

**5.** Line 357: To the statement

“However, NPFR is also expressed in non-neuronal tissues, raising the possibility that brain-derived NPF signaling targets non-neuronal cells.”

It is important to note that as far as I know (based on antisera and Gal4-drivers) NPF in the brain is only expressed in interneurons, so all targets of brain NPF neurons should reside within the brain or ventral nerve cord (VNC) where axons from descending NPF neurons project. NPFR outside the CNS should respond to hormonal NPF from gut EECs only. Thus it is not surprising if an ovarian NPFR is not involved. In any case the NPF targets of the CNS are yet to be identified.

**6.** Line 380: The screen for NPFR-expressing neurons that could mediate the ovarian response was not exhaustive, and the statement on line 407 a bit unfortunate: “Thus, we cannot exclude the possibility that 60G05-GAL4 driven NPFR depletion perturbs ovarian NPFR expression in an undetectable but significant manner and/or alters NPFR expression in cells that are yet to be described.”

This sentence also needs rephrasing while “in an undetectable but significant manner” sounds weird (you do detect a significant manner!).

**But importantly**, I would suggest considering a few putative NPFR-expressing cell systems that could mediate the NPF action to ovary cells.

***Strategy 1*** would be to use trans-tango labeling (or GRASP) to identify all NPF target neurons, since it appears that the NPRF-GAL4 lines are variable and incomplete.

***Strategy 2*** would be to screen other putative target cells that could express the NPFR.

The NPFR is functionally expressed on insulin-producing cells (IPCs) in the pars intercerebralis. ILPs could target the ovaries as hormone. Surprisingly the NPFR GAL4 lines used in the present study do not include the IPCs.

Does the 60G05-GAL4 show up in any neurons with axons reaching outside the CNS? It is hard to tell from the images provided. There are several types of neurons in the *abdominal ganglia* (in addition to Tdc-GAL4 neurons) that have efferent axons. Maybe some of these innervate the ovaries or have axon terminations in the periphery from which a hormonal substance could be released to reach the ovaries. For example neurons producing:

Pigment-dispersing factor,

Crustacean cardioactive peptide,

Ion transport peptide,

RYamide,

Allatostatin A,

Proctolin

ILP7

Also neurons expressing the ILP8 receptor Lgr3 (in abdominal ganglia) seem to innervate ovaries (Lgr3VP16-Gal4, from M Texada).

**To Discussion**

**7.** On Line 450: To the statement

“A most recent study showed that parental exposure to parasitoid wasps leads to depression of NPF-signaling in the adult fly brain, which triggers caspase-mediated germline apoptosis and ethanol preferences.”

Is this analysis of germ-line apoptosis any different from the one presented in the present paper? How is the regulation of ethanol seeking/preference coming about (what circuits/pathway)? Would be useful to get some more information here.

**8.** On Line 470: To the statement

“The recruitment of NPF-NPFR signaling requires parasitoid-specific visual and olfactory cues, and the NPF-responsive central region that integrates these sensory inputs is yet to be identified.”

Can the authors speculate which region is important? Commonly the so-called lateral horn (LH) of the brain is a target of pheromone-like signals and also receives visual input. In Fig. 5 one can see NPF expressing branches in the LH (upper left and right corners of image). Presumably both NPF cell types shown in Fig 5 have branches in the LH (and elsewhere).

**9.** On Line 473: It is not made clear to the readers that the study [42] describes a very different pathway.

“NPF expression in the wasp-exposed fly brain functions to modify oviposition preferences [5,17], whereas the mid-gut expressed NPF controls GSCs proliferation [42].”

Can the authors reveal under which conditions gut-derived NPF acts on ovaries to make clear it is very different from the wasp-induced signaling? The paper [42] describes NPF signaling induced by mating (which is a very different pathway).

**10.** On Line 476: To the statement

“Given the conserved function of NPF and its mammalian ortholog NPY in regulating stress responses [60–62], these questions are of fundamental relevance to both behavioral immunity and neuromodulation of germline physiology.”

This is too simplified. NPY/NPF also regulates feeding, metabolism, aggression, mating and clock functions, so one may also see an influence of altered NPF signaling on other behaviors and metabolism after exposure to a wasp. As the authors mention, stress may reallocate resources for ovary development (and fecundity) to somatic maintenance (like in diapause). Guess also sleep, feeding and receptivity to males would alter when a wasp appears. That would mean orchestration of behavior-metabolism-physiology for the purpose of protecting offspring (delaying offspring) when exposed to a wasp. This could mean that wasp-induced NPF targets many more systems than ovaries.

**11.** On Line 487: To the statement/conclusion

“For example, since proliferation and differentiation of adult stem cells is critical for tissue maintenance, including adult neurogenesis in mammals, it is intriguing to think that sensory inputs can modulate neuronal stem cell proliferation or differentiation. Although highly speculative at this time, if adult neurogenesis were to be continually modulated by sensory signals, then it would have broad implications for how we understand adult brain functions and maintenance.”

This is indeed speculative (but God’s ways are mysterious!).
